# Effects of COVID-19-Related School Closures on Student Achievement-A Systematic Review

**DOI:** 10.3389/fpsyg.2021.746289

**Published:** 2021-09-16

**Authors:** Svenja Hammerstein, Christoph König, Thomas Dreisörner, Andreas Frey

**Affiliations:** ^1^Goethe University Frankfurt, Frankfurt, Germany; ^2^Center for Educational Measurement, Faculty of Educational Sciences, University of Oslo, Oslo, Norway

**Keywords:** systematic review, COVID-19, school closure, student achievement, learning loss

## Abstract

The COVID-19 pandemic led to numerous governments deciding to close schools for several weeks in spring 2020. Empirical evidence on the impact of COVID-19-related school closures on academic achievement is only just emerging. The present work aimed to provide a first systematic overview of evidence-based studies on general and differential effects of COVID-19-related school closures in spring 2020 on student achievement in primary and secondary education. Results indicate a negative effect of school closures on student achievement, specifically in younger students and students from families with low socioeconomic status. Moreover, certain measures can be identified that might mitigate these negative effects. The findings are discussed in the context of their possible consequences for national educational policies when facing future school closures.

## Introduction

In spring 2020, the COVID-19 pandemic caused severe disruption to everyday life around the world. As one of several measures taken to prevent the spread of the virus, many governments closed schools for several weeks or months. Although school closures are considered to be one of the most efficient interventions to curb the spread of the virus (Haug et al., 2020), many educators and researchers raised concerns about the effects of COVID-19-related school closures on student academic achievement and learning inequalities. For instance, Woessmann (2020) estimated a negative effect of 0.10 *SD* on student achievement due to COVID-19-related school closures. Moreover, Haeck and Lefebvre (2020) estimated that socioeconomic achievement gaps would increase by up to 30%.

The negative effects of school closures due to summer vacation or natural disasters, and of absenteeism on student achievement are already well documented in the literature (for an overview see Kuhfeld et al., 2020a). Less is known, however, about the impact of COVID-19-related school closures on student achievement. The primary focus of the literature on COVID-19-related school closures to date was on the reception and use of digital learning technologies and remote learning (Andrew et al., 2020; Grewenig et al., 2020; Maity et al., 2020; Pensiero et al., 2020; Blume et al., 2021). Moreover, the psychological impact of COVID-19-related school closures, the use of school counseling in connection with COVID-19 (O'Connor, 2020; Xie et al., 2020; Ehrler et al., 2021; Gadermann et al., 2021; O'Sullivan et al., 2021), and the effects of the school closures on student motivation (Zaccoletti et al., 2020; Smith et al., 2021) were investigated. Existing projections of the impact of COVID-19 on student achievement paint quite a bleak picture. A learning loss of up to 38 points on the Programme for International Student Assessment (PISA^1^) scale is estimated, which corresponds to an effect size (Cohen's *d*) of 0.38 or 0.9 school years (Azevedo et al., 2020; Kuhfeld et al., 2020a; Wyse et al., 2020; Kaffenberger, 2021).

Thus, a year into the pandemic, it is a good time for a first stocktaking of the actual, evidence-based impact of COVID-19-related school closures on student achievement. Consequently, the present work aimed to answer two research questions. First, what was the general effect of COVID-19-related school closures in spring 2020 on student achievement in primary and secondary education? Second, did school closures have differential effects on specific student groups?

The review is organized following the reporting guidelines of the PRISMA statement (Page et al., 2021) and structured as follows. We first illustrate our systematic literature search, the inclusion criteria, the risk of bias assessment, and the synthesis of the relevant information from the studies selected. We then report the general and differential effects of the COVID-19-related school closures on student achievement, which are discussed in the context of their possible consequences for future national educational policies.

## Method

### Literature Search

**Figure 1:**
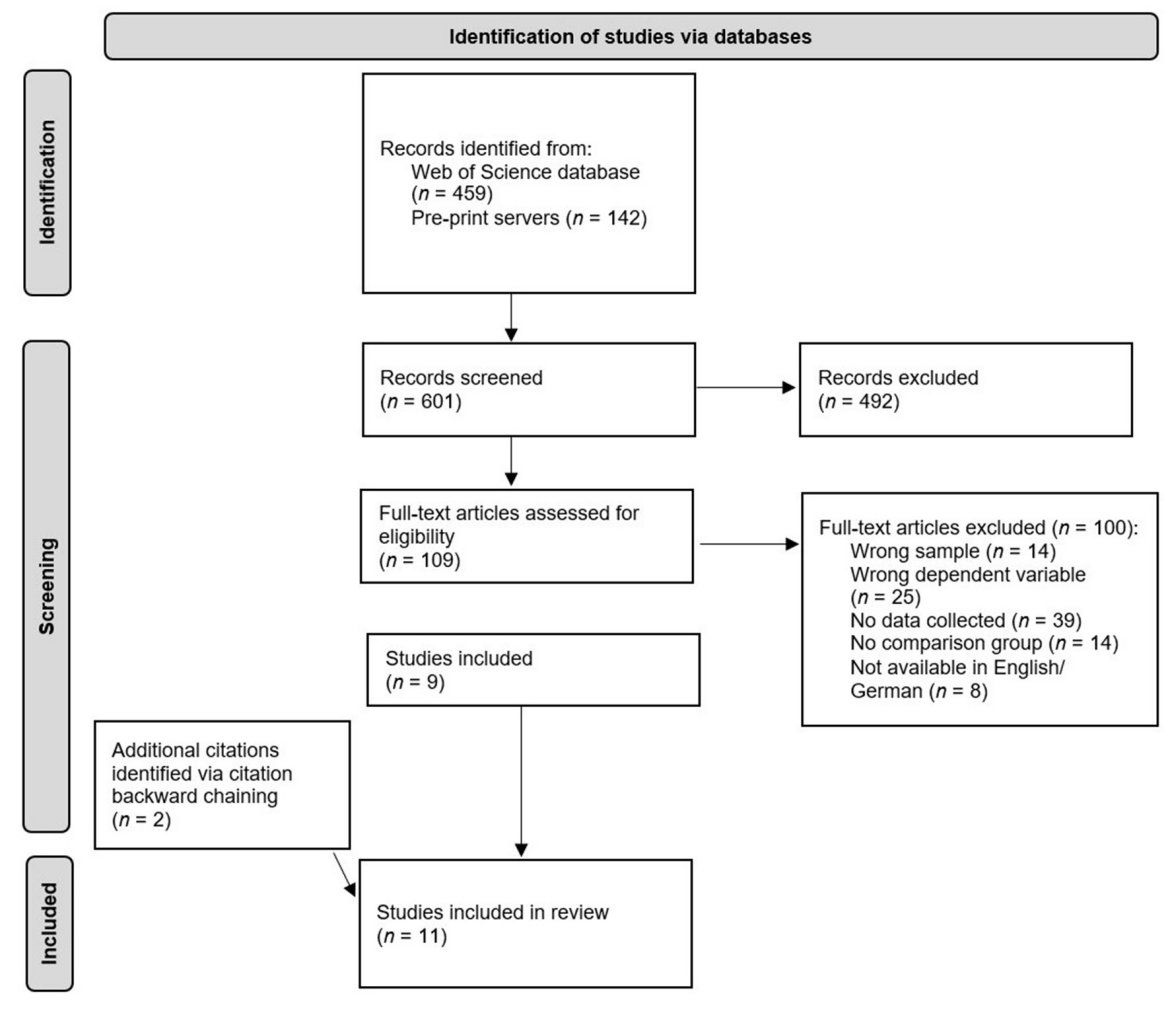
PRISMA flowchart of the literature search and screening process.

To identify relevant studies that investigated the effect of COVID-19-related school closures on student achievement, we searched the Web of Science database for articles published between March 1, 2020 and April 30, 2021. We used the following keywords and search string: [Covid OR Corona OR “SARS-CoV-2” AND school AND learn^*^ OR “test score” OR performance OR competenc^*^ OR achievement OR grades]. The results were refined by using the following categories: education, educational research, economics, education scientific disciplines, psychology educational, psychology multidisciplinary, social sciences interdisciplinary, and education special. The indexes searched were SCI-EXPANDED, SSCI, A&HCI, CPCI-S, CPCI-SSH, BKCI-S, BKCI-SSH, ESCI, CCR-EXPANDED, and IC. Because the COVID-19 pandemic was still ongoing at the time this review was written, and the field of research on the effects of COVID-19-related school closures on student achievement is rapidly evolving, we additionally searched the preprint servers PsyArXiv, EdArXiv, and SocArXiv using the aforementioned keywords. With this initial literature search, we obtained 601 potentially relevant studies. After selecting relevant articles out of these studies, we used the *backward reference searching* method (i.e., examining the works cited in the selected articles) to identify additional potentially relevant studies. See Figure 1 for a PRISMA flowchart of the literature search process.

### Selection of Studies

The abstracts of the studies selected were carefully read by the authors, and further inclusion was decided based on the following initial criteria. The studies (1) had to have a clear focus on COVID-19-related school closures, they (2) had to focus on primary and secondary education, and they (3) had to have student achievement (or test scores) as the dependent variable. This initial selection left 109 studies for potential inclusion in the review. These studies were thoroughly read by the authors and two research assistants. We carefully assessed the quality of included studies and based the decision to include studies in the review on the following primary set of inclusion criteria: Studies were required (1) to have collected actual data prior to and during/after COVID-19-related school closures, and (2) to have applied statistical analyses and to report an effect size. This set of inclusion criteria was chosen in order to select studies that provided the aforementioned evidence-based insights. Thus, reviews or discussions on how COVID-19 affects educational processes were excluded. Likewise, exploratory analyses or simple surveys (where only percentages were reported) were also excluded. For example, Chadwick and McLoughlin (2021) investigated the impact of COVID-19 related school closures on student's science learning. However, they only questioned teachers on the impact of COVID-19 related school closures on teaching, learning, and assessment. Because the study did not meet the inclusion criteria of having collected actual data on student achievement, including a comparison of data prior to and during/after COVID-19-related school closures, and applying statistical analyses rather than solely reporting percentages, the study was excluded from the systematic review. Similarly, studies by Haeck and Lefebvre (2020), Kaffenberger (2021), and Kuhfeld et al. (2020a) were excluded from the systematic review because they reported predicted effects of COVID-19 related school closures on student achievement but did not collect actual data prior to and during/after COVID-19-related school closures.

To determine the degree of rater agreement on the selection of the studies, a randomly selected subset of 20 studies was evaluated by both the authors and the research assistants. Any remaining divergent evaluations were highlighted in the evaluation forms and subsequently discussed. The second selection procedure yielded nine studies that were suitable for inclusion in the review. Subsequently, a backward search of references within the nine selected studies yielded two additional studies, which were then also included in the review.

### Risk of Bias Assessment

**Figure 2:**
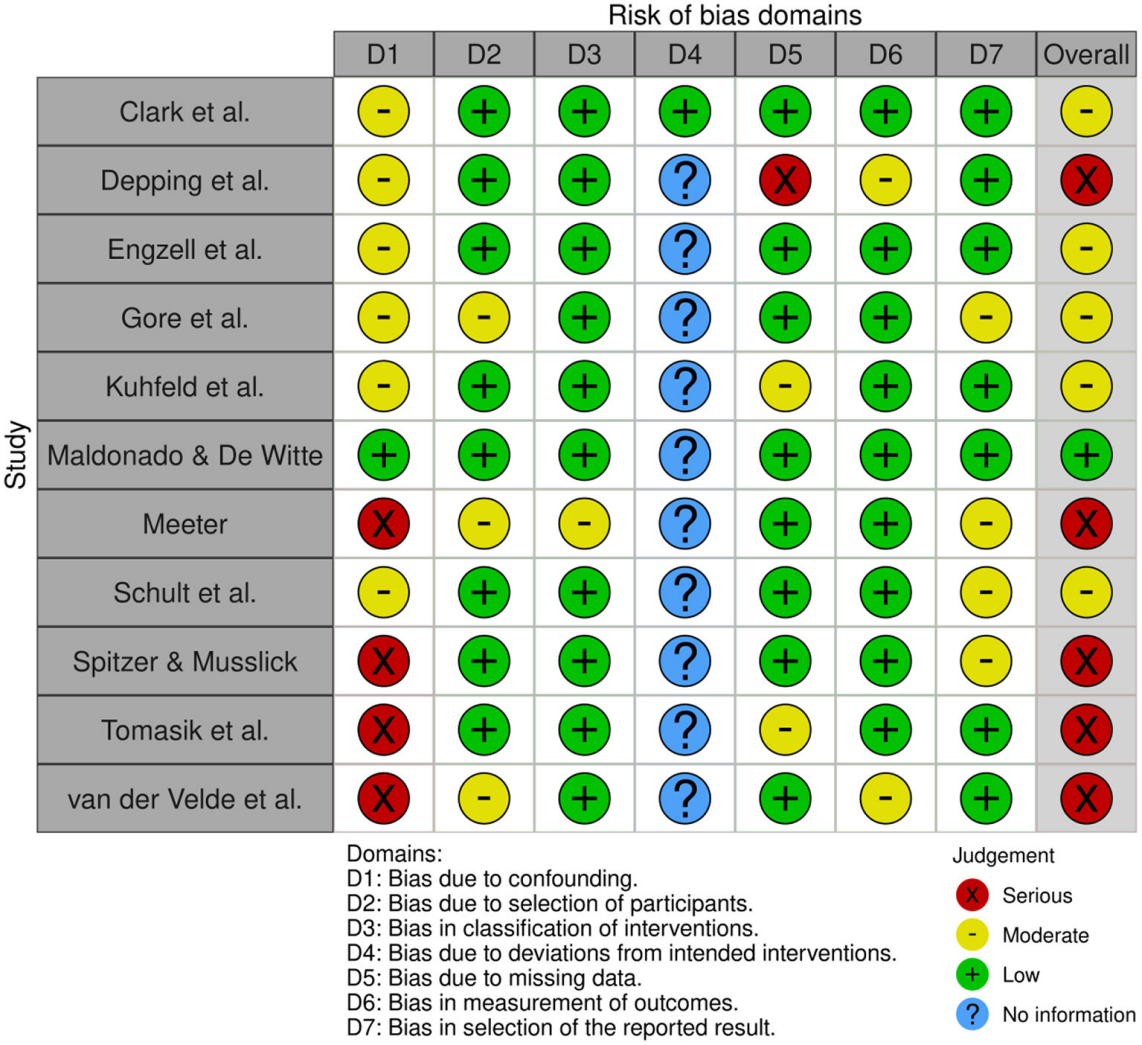
Result of the cochrane risk of bias assessment.

The Cochrane Risk Assessment of the included studies was conducted independently by the first and second author using the “Risk Of Bias in Non-Randomized Studies of Interventions” tool (ROBINS-I; Sterne et al., 2016). The result of the risk assessment is summarized in Figure 2. Taken together, the highest risk of bias is due to the lack of inclusion of potential confounding variables (Domain 1). Most studies, however, included at least a few relevant controls. Bias due to selection of participants was unlikely as the groups were formed naturally. Similarly, where applicable, interventions were classified correctly. Except for Clark et al. (2020), no information could be obtained about deviations from intended interventions. This is because the COVID-19 related school closures were not intended interventions. Thus, although there is no information, the risk due to deviations from intended interventions was deemed low. Lastly, bias due to missing data, measurement of outcomes, or selection of reported results is unlikely, as most studies exhibited very small proportions of missing data (except Depping et al., 2021), and were highly transparent in their reporting of the results (except, partially, van der Velde et al., 2021, who only report significant mean differences between groups in sufficient detail). Depping et al. (2021), however, thoroughly discuss and provide convincing reasons for assuming that missing data does not substantially influence their results.

### Synthesis

We synthesized the eleven studies by extracting the following information that was relevant for our research questions: (1) country, (2) duration of school closure, (3) sample description (type of school and sample size), (4) subjects for which student achievement was investigated, (5) statistical method, (6) general effects of the COVID-19-related school closures on student achievement, and (7) differential effects as reported by subgroup analyses (see Table 1 for a detailed list of the studies included). The focal piece of information was the reported general and differential effects. Where possible, general effects reported in different metrics (e.g., percentile scores), were converted to changes in *SD*. We then calculated the median of the reported effects, for the overall general effect, as well as for the general effect on reading and mathematics. In light of the relatively small number of studies, random- or even mixed-effects meta-analytic models were not feasible.

**Table 1:** Descriptive criteria of studies included.

**Authors(Country)**	**Duration of school closure**	**Type of school**	**Sample size**	**Subjects**	**Statistical method**	**General effect**	**Differential effects**
Clark et al.(China)	7 weeks	Secondary	1,835	Reading, Mathematics, English, Politics, History	DiD Regression	−0.22 *SD*	Larger effect in girls Larger effect in low-achieving students
Depping et al.(Germany)	8 weeks	Elementary and Secondary	~27,500	Reading, Mathematics	Difference Analyses	0 *SD* to +0.05 *SD* (reading)−0.03*SD* to −0.02 *SD* (mathematics)	—
Engzell et al.(Netherlands)	8 weeks	Elementary and Secondary	350,000	Reading, Mathematics	DiD Regression	−0.09 *SD* (reading)−0.14 *SD* (mathematics)	+60% learning loss in low-SES students
Gore et al. (Australia)	8 weeks	Elementary	>4,800	Reading, Mathematics	Linear Mixed Models	+0.04 *SD* (reading)+0.06 *SD* (mathematics)	Grade 3 in mathematics:−0.16 *SD* for low school-level SES, +0.15 *SD* for medium school-level SES
Kuhfeld, Ruzek, et al.(USA)	8 weeks	Elementary and Secondary	~7 Million	Reading, Mathematics	Change Score Analyses	−0.13 *SD* to−0.25 *SD* (mathematics)	Likely differences for different ethnicities
Maldonado and De Witte(Belgium)	7 weeks	Elementary	> 4,000	Reading, Mathematics, Social Sciences, Science, French	DiD Regression	−0.29 *SD* (reading)−0.19 *SD* (mathematics)	Larger effect in low-SES students
Meeter(Netherlands)	8 weeks	Elementary	~95,000	Mathematics	ANOVA	+0.20 *SD*	—
Schult et al.(Germany)	8 weeks	Secondary	~80,000	Reading, Mathematics	Change Score Analyses	−0.07 *SD* (reading)−0.09 *SD* to−0.03 *SD* (mathematics)	Larger effect on reading in high-performing students, larger effect on mathematics in low-performing students
Spitzer and Musslick (Germany)	8 weeks	Elementary and Secondary	> 2,500	Mathematics	Linear Mixed Models	−2.43% (error rate)	Larger improvements in low-achieving students
Tomasik et al.(Switzerland)	8 weeks	Elementary and Secondary	26,685	Reading, Mathematics	Growth Curve Models	−0.37 *SD* (elementary school)−0.10 *SD* (secondary school)	Larger effect in younger students, larger effect in high-performing students
van Der Velde et al.(Netherlands)	8 weeks	Secondary	133,450	French	Linear Mixed Models	+0.25 *SD* (correct solutions to open questions)	–

## Results

### General Effects of COVID-19-Related School Closures on Student Achievement

The studies on the effect of COVID-19-related school closures on student achievement selected for our review reported mixed findings, with effects ranging from−0.37 *SD* to +0.25 *SD* (*Mdn* = −0.08 *SD*). Most studies found negative effects of COVID-19 related school closures on student achievement. Seven studies reported a negative effect on mathematics (Clark et al., 2020; Kuhfeld et al., 2020b; Maldonado and De Witte, 2020; Tomasik et al., 2020; Depping et al., 2021; Engzell et al., 2021; Schult et al., 2021), five studies on reading (Clark et al., 2020; Maldonado and De Witte, 2020; Tomasik et al., 2020; Engzell et al., 2021; Schult et al., 2021), and two studies on other subjects, such as science (Maldonado and De Witte, 2020; Engzell et al., 2021). This is in line with expected learning losses due to COVID-19 related school closures and the assumption that, in spring 2020, the ad hoc implementation of online teaching gave students, teachers, schools, and parents little time to prepare for or adapt to measures of remote learning.

Three studies reported positive effects of COVID-19 related school closures on student achievement. Meeter (2021) and Spitzer and Musslick (2021) showed students to improve their mathematics achievement when learning with an online-learning software during the COVID-related school closures. Similarly, van der Velde et al. (2021) reported an increase in correct solutions on open questions within a French learning program. Interestingly, these three studies focused on online-learning software. Thus, the positive effects may be explained by the students under investigation being familiar working with the corresponding online-learning software prior to school closures. Hence, they did not have to adapt to a new learning environment when in-person teaching was interrupted due to COVID-19. Moreover, students increased the time using the online-learning software at home, were less distracted or experienced less time pressure in a home-schooling rather than classroom setting, or were presented with individualized assignments within the online program (see also Meeter, 2021; Spitzer and Musslick, 2021; van der Velde et al., 2021).

Additionally, two studies found positive effects on student achievement in mathematics and reading (Gore et al., 2021), or in reading only (Depping et al., 2021). This result might be accounted for by the achievement measurement being timed some months after school closures in both studies and the possibility of effective compensatory measures being implemented by teachers, schools, and local policy makers during this time to counteract learning losses, such as offering learning groups during summer vacation in parts of Germany (Depping et al., 2021).

Even though the median for the effect on mathematics and reading is comparable when averaging above all studies (*d* = −0.10 *SD* and−0.09 *SD* for mathematics and reading, respectively), some included studies found different effects for different subjects. On the one hand, reasons for finding larger learning losses in reading than in mathematics might be that “mathematics is easier to teach in distance learning, as it is simple to provide exercises and tests digitally or as worksheets” (Maldonado and De Witte, 2020, p. 13). As another explanation, many students might not speak the language in which they are tested in at home, hence, not benefitting much in their language skills during school closures (e.g., Maldonado and De Witte, 2020). On the other hand, reasons for finding larger learning losses in mathematics than in reading might be that students spent more time on reading during school closures and that supporting children in their reading skills might have been easier to realize for parents than supporting children in improving their competencies in mathematics (e.g., Depping et al., 2021; Schult et al., 2021).

### Differential Effects on Groups of Students

The studies selected for our review reported three main differential effects of COVID-19-related school closures on student achievement in different groups of students. First, the main finding was that younger children were more negatively affected in their learning than older children were (-0.37 *SD* vs.−0.10 *SD*; Tomasik et al., 2020). Second, children from families with a low socioeconomic status (SES) were more affected than children from families with a high SES were (Maldonado and De Witte, 2020; Engzell et al., 2021). In this context, one study reported an interaction between grade and SES, that is, for younger children from schools with low school-level SES, learning losses of 0.16 *SD* were found, while younger children from schools with medium school-level SES experienced learning gains of 0.15 *SD* (Gore et al., 2021). Third, low-performing students were more affected by COVID-19-related school closures in mathematics, while high-performing students were more affected by COVID-19-related school closures in reading (Schult et al., 2021). Finally, low-performing students benefited more from systematic online-learning methods (Clark et al., 2020; Spitzer and Musslick, 2021).

As the original studies were not designed to identify the reasons for these effects, additional studies are required to explain the three main differential effects exhaustively. In the following, we provide potential explanations as stated in the original studies. Regarding the first main differential effect (younger students are more affected compared to older students), Tomasik et al. (2020) state that the slower pace of students in primary school may be due to younger children relying more on cognitive scaffolding during instruction, because their capability for self-regulated learning might not be sufficiently developed. From a socio-emotional perspective, younger children might have been more sensitive to stressors related to the COVID-19 pandemic (Tomasik et al., 2020).

The reasons for students from low SES families being more affected relate to access to remote learning, their learning behavior, and the support provided from families and schools. Children from families with a low SES are less likely to have access to remote learning (UNESCO, 2021), are less often provided with active learning assistance from their schools (Tomasik et al., 2020), and spend less time on learning (Meeter, 2021) than children from families with a high SES. Moreover, parents with a high SES are more likely to provide greater psychological support for their children (OECD, 2019), which seems to be specifically relevant in a situation such as the COVID-19 pandemic.

The differential effect on low-performing and high-performing students may be due to high-performing students being capable of improving their performance regardless of the learning environment, while low-performing students specifically benefit from systematic online learning (Clark et al., 2020). Additionally, low-performing students might be less distracted in comparison to learning in a classroom setting (Spitzer and Musslick, 2021). Finally, with the possibility to adapt the assignments in online programs individually to the students, low-performing children might have been addressed more thoroughly according to their needs (Spitzer and Musslick, 2021).

## Discussion

The present work aimed to provide a first systematic overview of studies that reported effects of COVID-19-related school closures on student achievement and to answer two research questions. First, what was the general effect of COVID-19-related school closures in spring 2020 on student achievement in primary and secondary education? Second, did school closures have differential effects on specific student groups?

In sum, there is clear evidence for a negative effect of COVID-19-related school closures on student achievement. The reported effects are comparable in size to findings of research on summer losses (*d* = −0.005 *SD* to −0.05 *SD* per week; see also Kuhfeld et al., 2020a) and comparable to Woessmann's initial estimate. Hence, even though remote learning was implemented during COVID-19-related school closures, the effects achieved by remote learning were similar to those achieved when no teaching was implemented at all during summer vacation. Alarmingly, specifically younger children (Tomasik et al., 2020) and children from families with a low SES (Maldonado and De Witte, 2020; Engzell et al., 2021) were negatively affected by COVID-19-related school closures. This finding is in line with predictions of widening learning gaps and additive learning losses in subsequent school years (Grewenig et al., 2020; Haeck and Lefebvre, 2020; Pensiero et al., 2020; Kaffenberger, 2021). This indicates that most remote learning measures implemented during the first school closures in spring 2020 were not effective for student learning; there was no difference between them and the absence of systematic teaching during summer vacation.

However, the present review can also identify online-learning measures that seem to be beneficial for student learning. Taking a closer look at studies that reported positive effects of school closures on student achievement, three of these studies (Meeter, 2021; Spitzer and Musslick, 2021; van der Velde et al., 2021) used some kind of online-learning software to assess student achievement. Students in the studies of both Meeter (2021) and Spitzer and Musslick (2021) worked with online-learning software for mathematics, and students in the study of van der Velde et al. (2021) worked on online-learning software for language learning (i.e., for French). Hence, the positive effects of COVID-19-related school closures on performance in such online-learning programs may have occurred due to the increased use of software during school closures and the fact that students from these studies were familiar working with online-learning programs, hence, did not have to adapt to a new learning environment during COVID-19-related school closures. Additionally, Spitzer and Musslick (2021) reported that low-performing students benefited even more than high-performing students regarding their performance during COVID-19-related school closures from using the learning software. The authors explained this finding by considering that low-performing students were potentially less distracted by other students in a home-learning setting. These findings are in line with results by Clark et al. (2020), showing low-performing students to specifically benefit from systematic online material.

The present review gives insights into the effects of the COVID-19 related school closures on student achievement in spring 2020. It has to be noted that the number of countries for which evidence of these effects are available is still small, and clustered around developed countries. Especially studies from developing countries are not available yet. We know, however, that the reduction in in-person learning was smaller for low-income countries than for medium-income countries (UNESCO, 2021). Nevertheless, the proportion of students enrolled in primary or secondary education is considerably smaller in poorer countries (Ward, 2020). It may be possible that studies coming from developing countries provide novel insights into the general and especially the differential effects of the COVID-19 related school closures on student achievement. The results of our systematic review can serve as a benchmark for these studies, once they emerge in the literature.

The first COVID-19-related school closures in spring 2020 were followed by similar measures in the fall and winter of 2020/2021. Due to the cumulative nature of learning processes and student achievement, additional learning losses are likely. Nevertheless, school closures do not seem to be initiated as quickly now as they were at the beginning of the pandemic, which is positive for learning. To counter the learning losses, on a micro level, educational policy makers should determine potential supportive measures that increase the active learning time on task. On a macro level, national policy makers should determine potential compensatory measures to support students in their learning and to avoid failed educational careers. In this regard, systematic online material and software have been found to compensate for learning losses, specifically in high-risk children. Hence, educational policy makers and educators should be aware of the importance of providing children with systematic material and ensuring that high-risk children, in particular, have access to adequate learning environments in order to circumvent learning losses and widening learning gaps that may be caused by subsequent school closures. We expect future studies focusing on the subsequent school closures to provide a more differentiated picture of the effects of COVID-19 related school closures on student achievement. For instance, studies may investigate whether there are differences in educational outcomes across countries with differing lockdown measures. Similarly, studies may investigate the reasons for the subject-specific general effects and the three main differential effects identified in this systematic review. Such studies require longitudinal approaches, and may provide educational policy makers with crucial additional information.

The goal of this systematic review was to provide a first evidence-based insight into the effects of COVID-19-related school closures on student achievement in primary and secondary education. The onus is now on national educational policy makers to be aware of these effects and, together with educational and psychological research fields, to work toward the implementation of measures to mitigate or even counteract these negative effects. This may be one of the most important societal tasks for the post-COVID time.

## Data Availability Statement

The original contributions presented in the study are included in the article/supplementary material, further inquiries can be directed to the corresponding author.

## Author Contributions

SH and CK conducted the literature review and synthesis, with critical input by AF. SH wrote and revised the manuscript with contributions and feedback provided by CK, TD, and AF. All authors have been involved in the conceptual design of the review.

## Conflict of Interest

The authors declare that the research was conducted in the absence of any commercial or financial relationships that could be construed as a potential conflict of interest.

## Publisher's Note

All claims expressed in this article are solely those of the authors and do not necessarily represent those of their affiliated organizations, or those of the publisher, the editors and the reviewers. Any product that may be evaluated in this article, or claim that may be made by its manufacturer, is not guaranteed or endorsed by the publisher.

## Footnotes

^1^PISA is an international large-scale assessment testing the skills and knowledge of 15-year-old students in three core domains (Mathematics, Reading, and Science). Its main survey is administered every three years, with each rotating as the major domain. The results of the main surveys are scaled to fit approximately normal distributions, with means around 500 score points and standard deviations around 100 score points. A 10-point difference on the PISA scale corresponds to an effect size (Cohen's *d*) of 0.10 (OECD, 2019).
